# LINC00467: an oncogenic long noncoding RNA

**DOI:** 10.1186/s12935-022-02733-5

**Published:** 2022-10-07

**Authors:** Xuyu Chen, Qian Luo, Yanan Xiao, Jing Zhu, Yirao Zhang, Jie Ding, Juan Li

**Affiliations:** The Second Affiliated Hospital of Nanjing Medical University, Nanjing, 210011 China

**Keywords:** Long non-coding RNA, LINC00467, Cancer biomarker, Molecular mechanism, Pathway

## Abstract

Long non-coding RNAs (lncRNAs) have been found to play essential roles in the cell proliferation, fission and differentiation, involving various processes in humans. Recently, there is more and more interest in exploring the relationship between lncRNAs and tumors. Many latest evidences revealed that LINC00467, an oncogenic lncRNA, is highly expressed in lung cancer, gastric cancer, colorectal cancer, hepatocellular carcinoma, breast cancer, glioblastoma, head and neck squamous cell carcinoma, osteosarcoma, and other malignant tumors. Besides, LINC00467 expression was linked with proliferation, migration, invasion and apoptosis via the regulation of target genes and multiple potential pathways. We reviewed the existing data on the expression, downstream targets, molecular mechanisms, functions, relevant signaling pathways, and clinical implications of LINC00467 in various cancers. LINC00467 may serve as a novel biomarker or therapeutic target for the diagnosis and prognosis of various human tumors.

## Introduction

Cancer is a major public health problem with high global mortality and is the second leading cause of death in the United States. The latest research shows that in 2021, 1,898,160 new cancer cases and 608,570 cancer deaths are projected to occur in the United States, indicating a high mortality rate [1]. It is a while for scientific researchers to explore the potential mechanism how to regulate the cancers. Recently, more and more evidences show that lncRNAs have been found out some information of vital importance associated with cancers [2, 3]. The results of Human genome sequencing data have showed that less than 2% of the human genome have the function of protein-coding, and the other majority of genes do not have the ability to encode protein. By convention, the lncRNA do not have the potential to encode protein. But recently, increasing studies proved that a few of lncRNAs have the capacity to encode micro peptide [4–6]. LncRNAs are the RNA molecules that are longer than 200 nucleotides, linked to many human malignancies and aberrant lncRNAs expression can result in the development of diseases, especially cancer. These lncRNAs have been shown to promote or inhibit tumor progression via complicated pathways at the transcriptional and/or posttranscriptional levels, possibly due to their ability in chromosomal recycling, chromatin modification, DNA transcription, and mRNA and protein interactions [7]. For example, lncRNAs have been shown to act as both a source and an inhibitory regulator of microRNAs (miRNAs) [6, 8]. As shown in previous research, lncRNA HOTAIR functioned as a competing endogenous RNA to regulate HER2 expression by sponging miR-331-3p to regulate progression in gastric cancer [9]. Meanwhile, lncRNA HOTAIR/Sp1/miR-199a regulated cancer stemness and malignant progression in cutaneous squamous cell carcinoma [10]. Moreover, lncRNA also can bridges protein for modifying protein complexes to regulate cancers. Our group previous study explored that lncRNA CRNDE could directly bind with EZH2 to silence DUSP5 and CDKN1A expression to promotes colorectal cancer cell proliferation [11]. Furthermore, lncRNAs make a huge difference in regulating multiple processes of gene expression. LncRNAs can affect mRNA transcription, splicing, translation, export, import, and stability, thus causing phenotypic changes. In clinical terms, due to the specific expression, LncRNAs have been widely suggested as biomarkers and therapeutic targets, different from the conventional treatments [12–14].

**Fig. 1:**
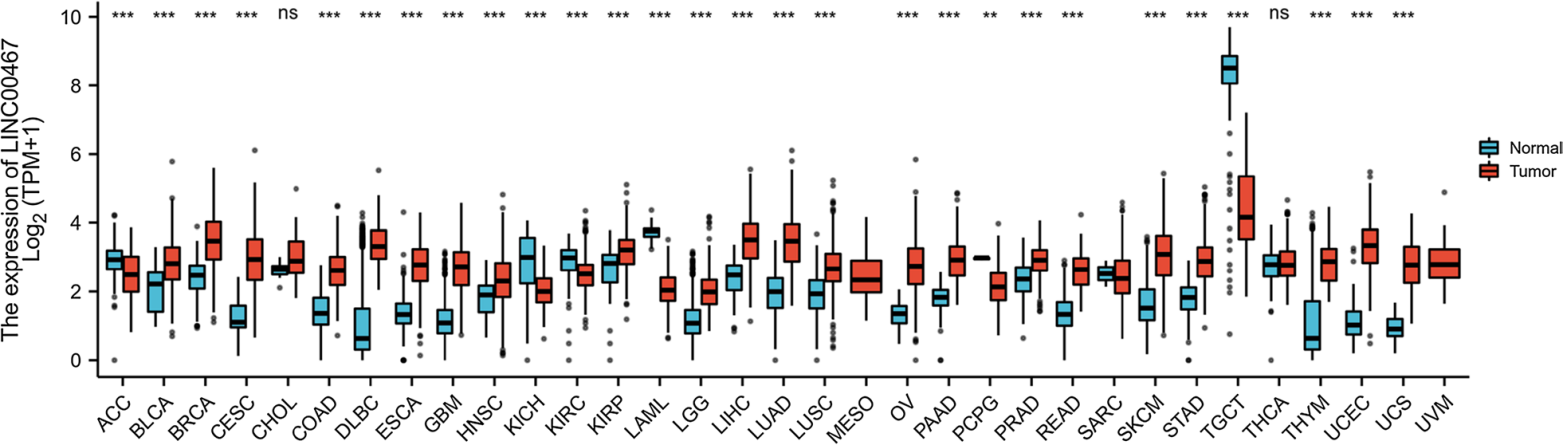
LINC00467 expression in various cancers

Long intergenic non-protein coding RNA 467 (LINC00467), also known as C1orf 97, is a recently discovered lncRNA [15]. LINC00467 has 4 transcripts and a full length of 3,508 bp, located in the 1q32.3 chromosome. Besides LINC00467 was found to be mainly localized to the nucleus [16]. The Cancer Genome Atlas (TCGA) database indicated that LINC00467 exhibits high pan-cancer expression across various cancers (Fig. 1). Studies have revealed that LINC00467 is associated with many clinical features and has been explored to be oncogenic. LINC00467 promoted tumor development and progression via several molecular and signaling pathways in different carcinomas including lung cancer, gastric cancer (GC), colorectal cancer (CRC), hepatocellular carcinoma (HCC), breast cancer, glioblastoma (GBM), head and neck squamous cell carcinoma (HNSCC), osteosarcoma. LINC00467 has also been known as a potential biomarker in the diagnosis, prognostication, and treatment of diseases. We still need to explore the precise molecular mechanism of LINC00467 in cancers. Furthermore, we also concluded the potential clinical target of LINC00467 and thereby providing from different angles.

## The role of LINC00467 in cancers

### LINC00467 in carcinomas

#### Lung cancer

Lung cancer is the most common malignant tumor, remained the leading cause of cancer death, with an estimated 1.8 million deaths. About 18% of cancer deaths are related to lung cancer [17]. Numerous risk predictions of lung cancer development are individuals age, smoking history, sex, personal and family history of cancer, and history of emphysema and chronic obstructive pulmonary disease. Among others, risk estimate include biomarker such as lncRNAs also act as an important part to lung cancer [18]. To lung cancer patients, the treatment of lung cancer is complex involving multiple treatment so that we need more precise therapeutic targets for these diseases.

Zhu et al. [19] analyzed the expression level of LINC00467 in lung cancer tissues and matched normal tissues in the Gene Expression Omnibus (GEO). They found that the expression of the LINC00467 was significantly upregulated in non-small cell lung cancer (NSCLC) and associated with advanced clinical stages and poor outcome [19].The analysis results of Gene Expression Profiling Interactive Analysis (GEPIA) show that LINC00467 was up-regulated in tumor tissues as compared to the normal tissues expressed [20]. They used qRT-PCR to identify LINC00467 expression in in different cell lines and also proved that LINC00467 was highly expressed in lung adenocarcinoma(LUAD) compared with human bronchial epithelial cells [20]. In addition, Ding et al. conducted the Kaplan–Meier survival analysis from the TCGA to explore the correlation between LINC00467 expression and the prognosis of NSCLC patients [21]. The Kaplan–Meier survival analysis showed that patients with high LINC00467 expression had shorter overall survival than patients with low LINC00467 expression [21]. RT‑qPCR assays were used to measure LINC00467 expression in 35 paired LUAD and adjacent normal lung tissues and researchers revealed that the expression level of LINC00467 in LUAD tissues was increased compared with that in adjacent normal lung tissues [22]. Wang et al. [23] analyzed online sequencing data from the TCGA LUAD cohort and observed a positive correlation between LINC00467 DNA copy number values and LINC00467 mRNA expression. Subsequently, they assessed the independent prognostic value of LINC00467 copy number alterations in terms of tumor metastasis and overall survival which predict poor prognosis in LUAD [23].The high expression of LINC00467 promoted the proliferation, migration, stemness, invasion of lung cancer cells and modulated cell cycle arrest and apoptosis [16, 19–22, 24]. These results suggested that LINC00467 played an oncogenic function and performed an important role in LUAD development.

**Fig. 2:**
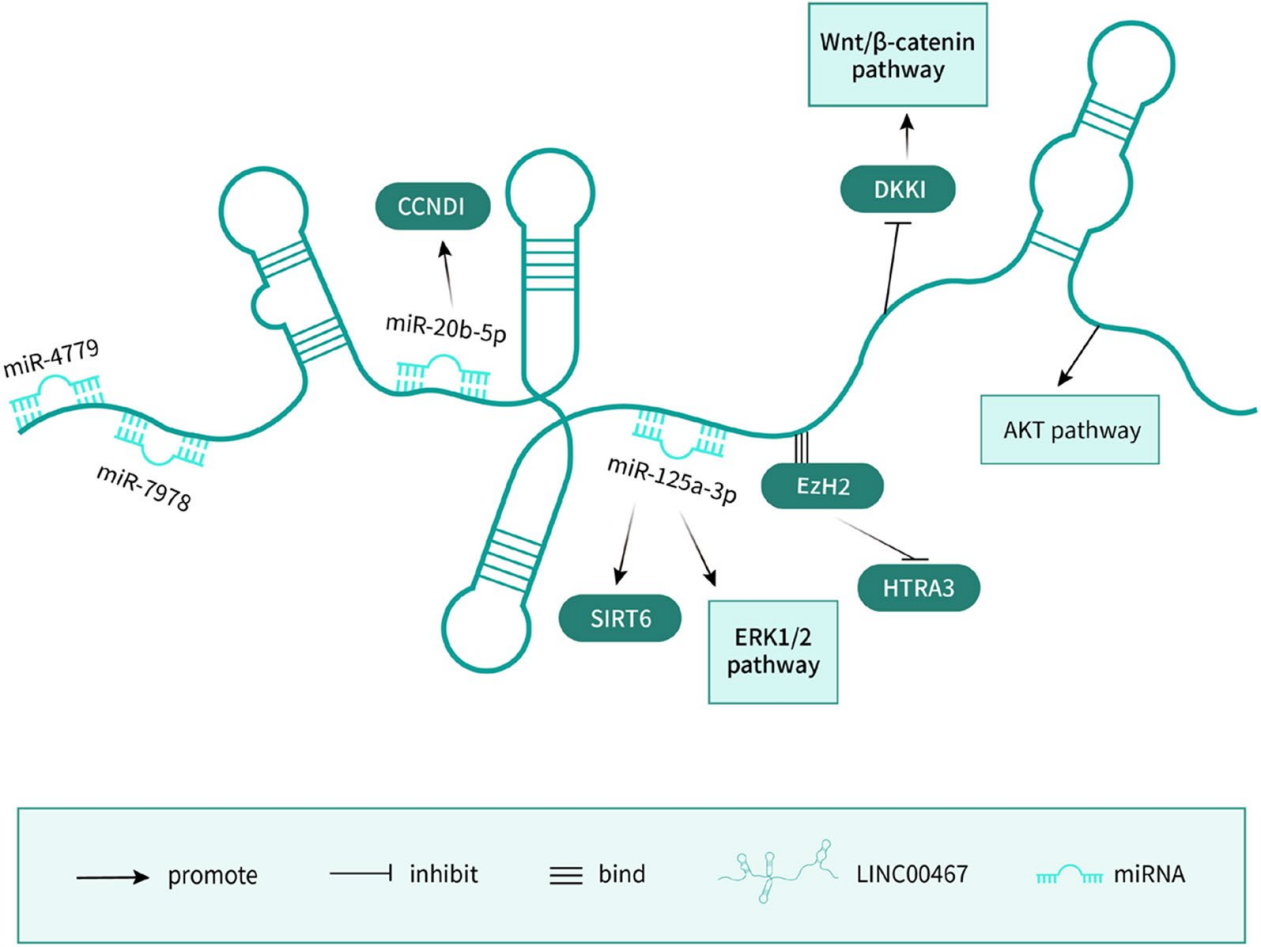
Mechanisms of LINC00467 in lung cancer. LINC00467 exerted its carcinogenesis function by sponging miR‐4779 and miR‐7978. A novel linc00467/miR-20b-5p/CCND1 signaling in LUAD. silencing LINC00467 upregulates miR-125a-3p to decrease cisplatin resistance via inhibiting SIRT6 and inactivating the ERK1/2 signaling pathway. LINC00467 bound EZH2 regulate HTRA3 to mediate oncogenic effects. STAT1-induced upregulation of LINC00467 leading to LUAD progression by epigenetically silencing DKK1 to activate Wnt/b-catenin signaling pathway. LINC00467 acts as a cancer promoter via activation of Akt signal pathway leading to advanced clinical stages and poor outcome

LINC00467 modulates multiple carcinogenic promotion via various molecular mechanism (Fig. 2). On the one hand, signaling pathways play a vital part in the process. Zhu et al. [19] demonstrated that LINC00467 acts as a cancer promoter via activation of Akt signal pathway leading to advanced clinical stages and poor outcome. Wang et al. [22] found that LINC00467 bound EZH2 regulate HTRA3 to mediate oncogenic effects. Yang et al. [16] determined that STAT1-induced upregulation of LINC00467 leading to LUAD progression by epigenetically silencing DKK1 to activate Wnt/b-catenin signaling pathway. On the other hand, Chang et al. [20]. Verified LINC00467 exerted its carcinogenesis function by sponging miR‐4779 and miR‐7978. Further research into the target genes of miR‐4779 and miR‐7978 will be required to fully elucidate the role of miR‐4779 and miR‐7978 in lung cancer. Ding et al. [21] revealed a novel linc00467/miR-20b-5p/CCND1 signaling in LUAD cells, providing efforts into lung cancer therapy. Wang et al. [23] constructed a competing endogenous RNA (ceRNA) network specific to LINC00467 in LUAD to regulate cancer progression. In addition, silencing LINC00467 upregulates miR-125a-3p to decrease cisplatin resistance via inhibiting SIRT6 and inactivating the ERK1/2 signaling pathway [24]. Taken together, these results indicate that LINC00467 acts as an oncogene in lung cancer and could represent a novel biomarker for its diagnosis, treatment, and prognosis.

#### Gastric cancer

Gastric cancer (GC) is a global health problem and it remains the third leading cause of cancer related death worldwide [25]. Eastern Asian nations are made up about half of gastric cancer diagnoses worldwide and it has more acutely effects on populations among developing nations [26]. Along with increased understanding of GC, many different therapies are emerging which may be more effective therapeutic options to GC.

According to the TCGA data, LINC00467 expression in 408 GC tissue is significantly higher than that compared with the paired adjacent 211 normal tissues, which suggested that LINC00467 plays an important role in GC development. Knockdown of LINC00467 can inhibit proliferation, migration, and invasion [27]. Highly expressed LINC00467 was linked with the tumor size, differentiation, N stage, and T stage in GC patients [28]. Besides, LINC00467 Played a role in various ways to regulate development in GC. LINC00467 could directly sponge miR-7-5p by acting as ceRNA to regulating EGFR signaling pathway in GC cells to contribute to tumorigenesis [27]. In addition, overexpressed LINC00467 enhanced the viability and proliferation but inhibited apoptosis of GC cells via raising ITGB3 level [28].

Many studies have shown that LINC00467 can provide a new research direction for molecular targeted therapy of GC. To influence the biological traits by regulating the expression of downstream proteins, LINC00467 may be 1 of the key factors.

#### Colorectal cancer

Colorectal cancer (CRC) remains a major health problem throughout the world, which puts enormous pressure on society. In the United States, an estimated 104,270 new cases of CRC occurred in 2021, and 52 980 deaths [1]. One of the greatest missions in the prevention of CRC is to discover effective drugs and the lncRNAs provide new ideas and issues at the same time.

QRT-PCR analysis evidenced that the expression levels of LINC00467 were positively upregulated in 31 CRC tissues compared with those in adjacent normal tissues. Knocking down LINC00467 inhibits the proliferation and induces apoptosis in HCT116 and HT29 cell lines by sponging miR‑451a as an endogenous competitor [29]. Data from GEO and GEPIA databases indicated that high LINC00467 expression levels correlated with poor overall survival (OS) and recurrent-free survival (RFS) rates [30]. Additionally, Li et al. [31] found that LINC00467 competes with Ferritin Light Chain (FTL), one subunit of ferritin, is capable to promote the growth of cancer cells, including HeLa cells and glioblastoma cells, for binding miR-133b to regulate chemoresistance and metastasis of colorectal cells. Last but not least, Wang et al. [32] revealed that LINC00467 has the ability to encode proteins, as mentioned earlier in the introduction. LINC00467 encodes micro peptide ATP synthase–associated peptide (ASAP) to promotes colorectal cells progression by directly modulating ATP synthase activity.

#### Hepatocellular carcinoma

Hepatocellular carcinoma (HCC) is a leading cause of cancer-related death and its incidence is growing worldwide [33]. Cirrhosis is the leading cause of HCC death in patients with and Alcohol-induced cirrhosis accounts for 15–30% of the disease [34]. In addition, improved strategies using new biomarkers (for example, long intergenic non-protein coding RNA) might cure patients with HCC disease.

The data from TCGA evidenced that higher expression of LINC00467 in HCC samples compared with the normal samples. RT-qPCR confirmed the upregulated level of LINC00467 in HCC cell lines than normal liver cells [35], revealing that LINC00467 possibly play an oncogenic effect in HCC procession. Next, overexpression of LINC00467 was positively correlated with tumor size and vascular invasion and enhances cell proliferation, cell cycle progression and migration, with HCC cell apoptosis reduced in vitro. Besides, LINC00467 expression was also significantly associated with poor overall survival of patients, which proved that overexpression of LINC00467 is linked with many poor prognosis of HCC patients [36].

LINC00467 functioned as carcinogenic promotion factor via numerous molecular mechanisms. In exploring the downregulation mechanisms, Jiang et al. [37] found that the gene for TRAF5 was revealed to be a neighboring gene of LINC00467 in the genome in database and qRT‐PCR and western blot analysis showed that LINC00467 downregulated both mRNA and protein expression of TRAF5. Then researchers used RNA pull‐down and RIP assays further to confirm that LINC00467 could bind with IGF2BP3 to promote the TRAF5, promoting cell proliferation and metastasis in HCC [37].LINC00467 not only can bind proteins come into play, but also can sponge with mRNA to regulate cancer development. Wang et al. [38] suggested that LINC00467 inhibited NR4A3 expression via interacting with NR4A3 mRNA to play its oncogenic roles.

Additionally, Li et al. [35] found that Axitinib treatment silenced LINC00467 can both inhibit proliferation and invasion of HCC cells. They found a negative correlation between miR-509-3p and LINC00467 in HCC cancer. Moreover, they performed the RIP assay and the luciferase reporter assay to confirm the direct interaction between LINC00467 and miR-509-3p. They further verified that PDGFRB was a downstream target of miR-509-3p [35]. Some molecular mechanisms like above, the LINC00467/miR‐18a‐5p/NEDD9 axis and the LINC00467/miR-9-5p/PPARA axis also have been convinced of being involved in tumorigenesis of HCC [36, 39].

#### Breast cancer

Breast cancer (BC) is the most common cancer among women and is the main leading cause of cancer death worldwide [40]. Surgical excision and chemotherapy are still the main options for clinical treatment. Early detection strategies, access to early diagnosis and better access to effective treatments can be a large advantage for patients diagnosed with breast cancer [41]. It is crucial to explore new biomarkers that can strengthen the diagnosis and treatment of BC, and also can improve its prognosis.

The expression level of LINC00467 in BC tissues and cells is obviously upregulated compared with paired adjacent tissues and normal breast epithelial cells. Functionally, several research revealed that silencing LINC00467 could suppress BC cell proliferation, migration and invasion [42, 43]. Mechanically, LINC00467 down-regulated miR-138-5p and protein level of lin-28 homolog B (LIN28B) via a direct interaction to serve as a potential target [43]. In addition, Bo et al. [42]. explored more mechanisms that high expression of LINC00467 driven by DNA methylation is associated with the protein TGF-β2 expression and LINC00467 correlated with the infiltration of CD4 + and CD8 + T cells suggests that LINC00467 was likely to inhibit anti-tumor immunity.

#### Glioblastoma

Glioblastoma (GBM) is a common brain cancer which can happen anywhere in the central nervous system [44]. GBM has an annual incidence of 3–5 per 100,000 people [45]. Despite recent progress in surgical, chemotherapeutic, and radiotherapy methods, patient prognosis remains poor. Therefore, further study is needed to elucidate more detailed mechanisms.

Gao et al. [46] revealed the high expression of LINC00467 in glioma cell lines through qRT-PCR analysis. Subsequently, reduced expression of LINC00467 could decrease the proliferation, migration, and invasion of GBM cells through CCK-8 assay, the cell migration assay, and the cell invasion assay. The luciferase reporter assay and the RIP assay results indicated that miR-200a could bind to LINC00467. Analysis using the the online tool starBase prediction database demonstrated that E2F3 was identified as a potential target for miR-200a [46]. Another study found that the patients with higher levels of LINC00467 showed advanced tumor stage and poor overall survival. Functionally, several research also revealed that silencing LINC00467 could suppress GBM cell proliferation, invasion and migration but induce cell apoptosis. Mechanically, LINC00467 inhibits the expression of P53 by interacting with the factor DNMT1, thus leading to abilities of proliferative and invasives in GBM [47]. Furthermore, LINC00467 upregulate IP6K2 expression via sponging of miR-339-3p to modulate GBM proliferation and cell apoptosis [48]. In addition, researchers found that LINC00467 also can promotes breast cancer progression by sponging of miR-485-5p [49]. All researches suggest that LINC00467 can be a potential oncogene target of GBM. It would be experimental basis for clinical prognosis judgments and targeted therapy of this tumor.

#### Head and neck squamous cell carcinoma

Head and neck squamous cell carcinoma (HNSCC) is a highly heterogeneous malignant tumor with high mortality [50], and the 5-year survival rate is only 40–50%[51]. Further study is needed to evidence more detailed mechanisms.

In HNSCC tissues and cells, the expression of LINC00467 has been demonstrated to upregulated compared with normal tissues and cells indicating that it may serve as an oncogene in HNSCC. Highly expression of LINC00467 is correlated with several cell functions, including proliferation, invasion, apoptosis. LncATLAS website showed that Linc00467 mainly located in the cytoplasm and Further study confirmed that TFAP2A was a downstream target of miR-1285-3p through the luciferase reporter assay [52].

Additional study using a wound healing assay measured cell migration in transfected cells suggested that knockdown of LINC00467 can promote HNSCC cell migration. Western blot analysis suggested that LINC00467 knockdown resulted in a marked increase in E-cadherin protein levels, but decreased N-cadherin protein levels. These data suggest that LINC00467 can facilitate cell migration and invasion. Mechanically, miR-299-5p was confirmed to can sponge with LINC00467, indicating that it acts as a ceRNA for this miRNA. The USP48 as downstream target protein of miR-1299-5p exerts an oncogenic effect in HNSCC regulated by LINC00467 [53].

#### Other cancers

In addition to the various carcinomas mentioned above, LINC00467 is relevant in other carcinomas. In Esophageal squamous cell carcinoma (ESCC), LINC00467 acted as an oncogene in ESCC by enhancing cell proliferation and preventing cell apoptosis via miR‐485‐5p/DPAGT1 axis [54]. In testicular germ cell tumors (TGCT), LNC00467 was positively linked with the poor prognosis and pathological grade of TGCT. Furthermore, LNC00467 could promote the migration and invasion of TGCT cells by regulating the expression of AKT3 and influencing total AKT phosphorylation [55]. In prostate carcinoma (PC), LINC00467 promotes PC progression via the miR-494-3p/STAT3 axis [56]. In Cervical Cancer, LINC00467 functioned as a competing endogenous RNA against miR-107 to suppress KIF23, resulting in induced cell migration, invasion, and epithelial-mesenchymal transition (EMT) in vitro [57]. In bladder cancer, LINC00467 is highly expressed in bladder cancer and can facilitate the progression of bladder cancer via regulating the NF-kB signaling pathway [58]. In acute myeloid leukemia (AML), LINC00467 upregulated oncogene SKI expression by sponging miR-339. Thus, the LINC00467/ miR-339/SKI pathway possibly has important functions in tumorigenesis of AML [59].

### LINC00467 in sarcomas

#### Osteosarcoma

Osteosarcoma is the most common primary bone cancer in children and young adults [60]. With high morbidity and mortality despite accomplishment of diverse therapeutic modalities [61].The role of lncRNA in osteosarcoma is an area that is clearly under active investigation with hopes of improving the treatment of the disease [62].

Compared with paired adjacent tissues and normal osteosarcoma cells, LINC00467 expression in osteosarcoma tissues and cells is obviously upregulated. Functionally, high levels of LINC00467 could promote osteosarcoma cell proliferation, invasion, migration and EMT but induce cell apoptosis. Furthermore, The Kaplan–Meier survival analysis showed that the high expression of LINC00467 was associated with poor overall survival rate in osteosarcoma patients. Researchers used qRT-PCR to find the subcellular location of LINC00467, and the results revealed that LINC00467 was predominately located in the cytoplasm rather than cell nucleus [63, 64].

Mechanistically, MA et al. [63] verified the binding site between LINC00467 and miR‐217 through the luciferase reporter assay. To further investigate the downstream molecular mechanism of miR‐217, they used starBase database to predict the target gene of miR‐217 and found that HMGA1 was a candidate target gene of miR‐217 and subsequent experimental results also confirmed this prediction. They concluded that LINC00467 promoted the progression of osteosarcoma by regulating the miR‐217/HMGA1 axis [63]. YAN et al. also explored that miR‑217 resulted in the downregulation of KPNA4 in osteosarcoma cells [64].

## Conclusions

In recent years, as direct Genome-wide association sequencing technologies advanced, a large number of lncRNAs have been identified associated with various types of cancer. LncRNAs may play tumor-suppressive and oncogenic functions [65]. In this review, we summarize the findings of LINC00467 current exploration. Many studies have verified that LINC00467 is highly expressed in a variety of carcinoma and sarcoma tissues and cells. LINC00467 confirmed as a crucial role to regulate tumor cells proliferation, invasion, migration, apoptosis and other aspects. (Table 1). As an emerging lncRNA in recent years, LINC00467 was found to be up-regulated in 14 cancers. LINC00467 often functions as a potential oncogene and shows great promise in the field of tumor exploration and treatment. There was a substantial relationship between the highly expressed LINC00467 and the clinicopathological characteristics, including tumor size, TNM stage, tumor grade and distance metastasis.

**Table 1:** LINC00467 with tumor type, clinical significance and functions in cancers

Tumor	Tumor type	Clinical significance	Function	Refs.
Lung cancer	carcinomas	Tumor size, TNM stage, Distant metastasis, Poor overall survival, DDP chemoresistance	Proliferation, migration, invasion, apoptosis, stemness	[16, 19–23, 24]
Gastric cancer	carcinomas	Tumor size, TNM stage,	Proliferation, migration, invasion, apoptosis	[27, 28]
Colorectal cancer	carcinomas	Tumor‑Node‑Metastasis stage, Poor overall survival and recurrent free survival, 5‐fluorouracil‐based chemoresistance	Proliferation, migration, invasion, apoptosis	[29–31, 32]
Hepatocellular carcinoma	carcinomas	Tumor size, Tumor size, Vascular invasion, Overall survival, Lymphatic metastasis, Axitinib resistance	Proliferation, migration, invasion, apoptosis	[35–38, 39]
Breast cancer	carcinomas	Overall survival, Disease specific survival, Disease free survival, Progression free survival, Tumor metastasis and poor prognosis, EMT	Proliferation, migration, invasion	[42, 43]
Glioblastoma	carcinomas	Overall survival, Tumor grade	Proliferation, migration, invasion, apoptosis	[46–48, 49]
Head and neck squamous cell carcinoma	carcinomas	EMT	Migration, invasion, apoptosis	[52, 53]
Esophageal squamous cell carcinoma	carcinomas	Not mentioned	Proliferation, apoptosis	[54]
Testicular germ cell tumors	carcinomas	Not mentioned	Migration, invasion	[55]
Prostate carcinoma	carcinomas	Not mentioned	Proliferation, migration, invasion, apoptosis	[56]
Cervical cancer	carcinomas	Overall survival, EMT	migration, invasion	[57]
Bladder cancer	carcinomas	Disease-free survival	Migration, invasion	[58]
Acute myeloid leukemia	carcinomas	Overall survival	Proliferation, migration, invasion, apoptosis	[59]
Osteosarcoma	sarcomas	Tumor size, TNM stage, EMT, Distant metastasis, Anatomic location, Age at diagnosis, Overall survival	Proliferation, migration, invasion, apoptosis	[63]

**Fig. 3:**
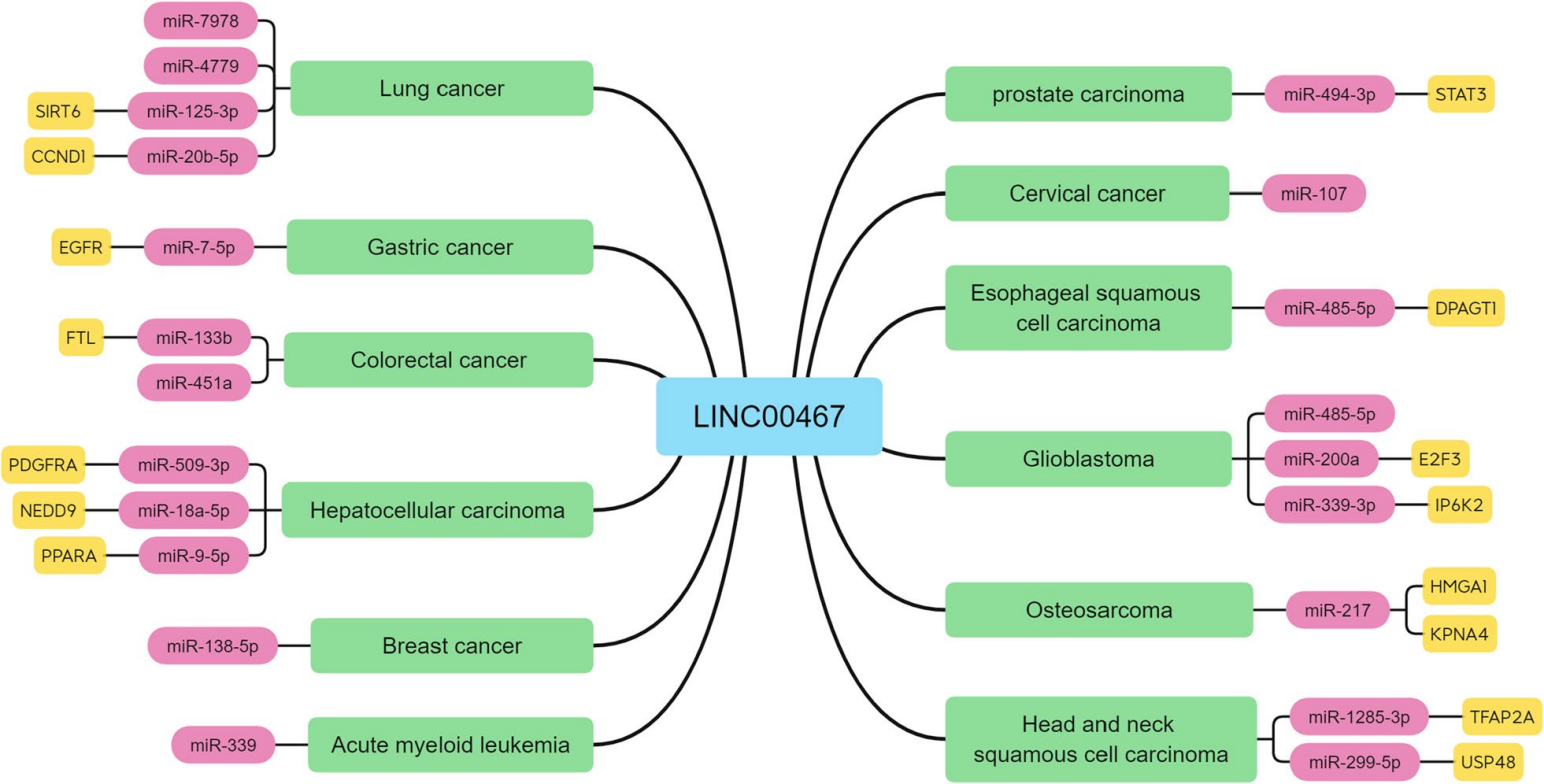
In 12 human cancers, the ceRNA network centered on LINC00467 involves the regulation of 20 miRNAs and 15 mRNAs

**Fig. 4:**
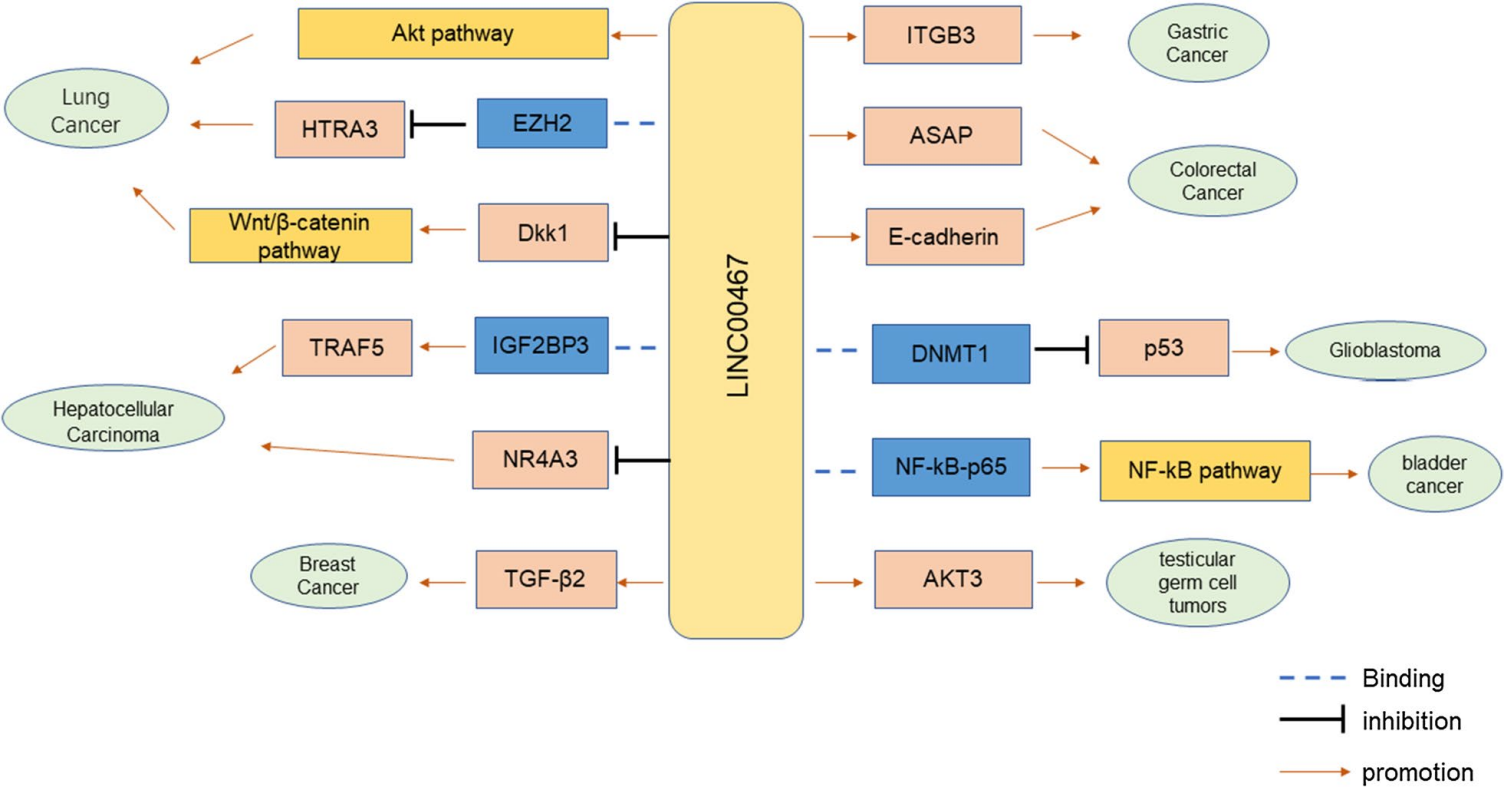
Role of LINC00467 in carcinogenic signaling pathways

The underlying mechanisms of LINC00467 are expressed in many forms (Table 2). LINC00467 established a complex ceRNA network by competitively binding 20 miRNAs in 12 cancers (Fig. 3). LINC00467 can also directly regulate downstream protein-coding genes, thereby promoting the occurrence and development of breast cancer via TGF-β2, lung adenocarcinoma via EZH2, and gastric cancer via ITGB3, etc. In addition, LINC00467 was found to play a regulatory role in the NF-κB signaling pathway, Wnt signaling pathway and AKT signaling pathway (Fig. 4). Under stress conditions, additional molecules may affect elements of the LINC00467-target genes axis, making it challenging to explore the specific regulatory mechanism of LINC00467.

**Table 2:** Summary of the mechanism studies of LINC00467 in cancers

Tumor	Expression	Downstream targets	Mechanism	Refs.
Lung cancer	up	miR-4779 miR-7978	Regulating miR-4779 Regulating miR-7978	[20]
		Akt	Regulating Akt signaling pathway	[19]
		HTRA3	Regulating HTRA3 expression via EZH2	[22]
		CCND1	Sponging miR-20b-5p to regulate CCND1	[21]
		SIRT6	Sponging miR-125a-3p to regulate SIRT6 and ERK1/2 pathways	[24]
		Dkk1	Targeting Dkk1 to regulate Wnt/β-catenin pathways	[16]
Gastric cancer	up	EGFR	Sponging miR-7-5p to regulate EGFR	[27]
		ITGB3	Regulating ITGB3	[28]
Colorectal cancer	up	FTL	Sponging miR-133b to regulate FTL	[31]
		miR-451a	Regulating miR-451a	[29]
		ASAP	Regulating ASAP	[32]
		E-cadherin	Regulating E-cadherin	[30]
Hepatocellular carcinoma	up	PDGFRA	Regulating miR-509-3p/PDGFRA axis	[35]
		TRAF5	Targeting to IGF2BP3 regulate TRAF5	[37]
		NR4A3	Regulating NR4A3	[38]
		NEDD9	Regulating miR-18a-5p/NEDD9 axis	[36]
		PPARA	Regulating miR-9-5p/PPARA axis	[39]
Breast cancer	up	miR-138-5p, Lin28b	Regulating miR-138-5p Regulating Lin28b	[43]
		TGFβ2	Regulating TGFβ2	[42]
Glioblastoma	up	miR-485-5p	Regulating miR-485-5p	[49]
		P53	Targeting to DNMT1 to regulate P53	[47]
		E2F3	Sponging miR-200a regulate E2F3	[46]
		IP6K2	Regulating miR-339-3p/IP6K2 axis	[48]
Head and neck squamous cell carcinoma	up	TFAP2A	Regulating miR-1285-3p/TFAP2A axis	[52]
		USP48	Regulating miR-299-5p/USP48 axis	[53]
Esophageal squamous cell carcinoma	up	DPAGT1	Regulating miR-485-5p/DPAGT1 axis	[54]
Testicular germ cell tumors	up	AKT3	Regulating AKT3	[55]
Prostate carcinoma	up	STAT3	Regulating miR-494-3p/STAT3 axis	[56]
Cervical cancer	up	miR-107	Regulating miR-107	[57]
Bladder cancer	up	NF-kb-p65	Targeting NF-kB-p65 to regulate NF-kB signaling pathway	[58]
Acute myeloid leukemia	up	SKI	Regulating miR-339/SKI axis	[59]
Osteosarcoma	up	HMGA1	Regulating miR-217/ HMGA1 axis	[63]
		KPNA4	Regulating miR-217/ KPNA4 axis	[64]

As a novel lncRNA, LINC00467 has the unique feature. LINC00467 possesses a coding ability that most other lncRNAs do not have. Ge et al. [32] explored that LINC00467 could encode a micropeptide called ASAP, inhibiting the progression of colorectal cancer. This discovery changes traditional knowledge framework and provides a novel idea for new cancer drugs.

The central dogma of molecular information flows information from gene to RNA to protein [66]. The mechanisms occur mainly at both the transcriptional and post-translational levels. We forecast that more underlying mechanisms are remained to be identified, such as variable shear, A-to-I editing [67, 68], m6A methylation[69; 70] at RNA levels and glycosylation [71], methylation [72], phosphorylation [73], acetylation [74], ubiquitination [75] in protein levels. From different perspectives, more studies will be conducted to explore more mechanisms. Ideally, these methods will be applicable to whole-gene studies so as to allow future researches more systematic and precise.

## Perspectives

**Fig. 5:**
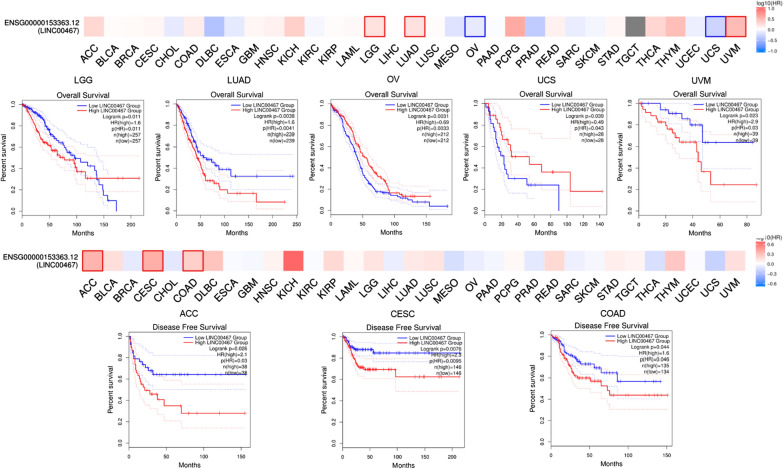
Relationship between LINC00467 expression level and patient survival in various tumors using GEPIA2

LINC00467 may have great translational value in clinical studies. GEPIA2 datasets were used to study the correlation between LINC00467 expression and prognosis of different tumor patients (Fig. 5). The figure displays a close connection between the expression of LINC00467 and prognosis of various cancer. In addition, LINC00467 is found to have the potential to increase tumor cell resistance to multiple chemotherapeutic agents. For example, downregulating the expression of LINC00467 could contribute to inhibiting cell sensitivity to Axitinib in hepatocellular carcinoma and regulate CRC cell resistance against 5-FU treatment [31]. These findings may provide a new insight for the development of tumor treatment in future. More clinical studies are needed to confirm the influence of LINC00467 on tumor drug resistance.

Currently, our knowledge of LINC00467 is very limited, and this lncRNA has not been thoroughly explored in cancers. Recent studies show that lncRNA can function not only as an oncogene but as a tumor suppressor at the same time in different cancers. MALAT1 has previously been described as an oncogene to promote metastasis across various cancers while recent studies showed that overexpression of MALAT1 suppresses the metastatic ability of breast cancer cells [76]. So, whether LINC00467 can act as an oncogene biomarker in wide range of cancer types is still uncertain. We still need a lot of basic experiments to perfect the function and role of LINC00467.

Cancers tend to be diagnosed at advanced stages and the chance of cure is relatively low. Recent researches discover many potential cancer biomarkers, but few transition to clinical applications. The expression levels of LINC00467 in various body fluids, including whole blood, plasma, urine, saliva, and gastric juices have not been clearly verified. As next generation sequencing and mass spectrometry techniques continue to evolve, more and more lncRNAs have been explored to hold a huge potential for gene-based therapy. Choosing medicines that target just one gene is insufficient since cancer is influenced by complicated networks. Researchers can also use the combined detection method to obtain better diagnostic value which involves combining genes with various traditional diagnostic markers. In addition, even if there is a perfect target, it is difficult to obtain an ideal delivery method. The tumor microenvironment is complex, so lncRNAs delivery and application are very difficult. The combination of multiple vectors, such as nanoparticles binding to tissue-specific receptors, could be an option to facilitate precise delivery of lncRNAs.

As a whole, high expression of LINC00467 has been significantly associated with cancer prognosis but applied to clinical treatment are still a long way to explore on account of whether all tumors high expression levels of LINC00467 are ideal targets for diagnosis and therapy remains unknown. Further studies are needed to confirm this biological factor expression stably in various cancers and substances stably expressed in bodily fluids have strong potential in the diagnosis of disease. In this review, we reported on the expression and molecular mechanisms of LINC00467 in various cancers. With the rapid progress of medical technology, the relationship between LINC00467 and various tumors can be verified completely, which will provide a theoretical basis for the targeted treatment of LINC00467.

## Data Availability

Not applicable.

## Abbreviations

lncRNA: Long non-coding RNA
LINC00467: Long intergenic non-protein coding RNA 467
ceRNA: Competing endogenous RNA
miRNA: MicroRNA
TCGA: The cancer genome atlas
GEO: Gene expression omnibus
NSCLC: Non-small cell lung cancer
LUAD: Lung adenocarcinoma
GEPIA2: Gene expression profiling interactive analysis 2
GC: Gastric cancer
CRC: Colorectal cancer
HCC: Hepatocellular carcinoma
BC: Breast cancer
GBM: Glioblastoma
HNSCC: Head and neck squamous cell carcinoma
ESCC: Esophageal squamous cell carcinoma
TGCT: Testicular germ cell tumors
PC: Prostate carcinoma
AML: Acute myeloid leukemia
EMT: Epithelial-mesenchymal transition
OS: Overall survival
RFS: Recurrent-free survival
FTL: Ferritin light chain
ASAP: ATP synthase–associated peptide
